# Fracture of the lateral process of the talus with associated deltoid ligament injury: a report of 2 cases

**DOI:** 10.1186/s12893-022-01781-y

**Published:** 2022-10-04

**Authors:** Xiang-Yun Jin, Wei-Yuan Xiao, Tao He, Yu-Qi Dong, Chao Zhang

**Affiliations:** Department of Orthopedics, Renji Hospital, School of Medicine, Shanghai Jiaotong University, Shanghai, 200127 People’s Republic of China

**Keywords:** Ankle, Fracture, Talus, Trauma, Case report

## Abstract

**Background:**

Fractures of the lateral process of the talus (LTPF) are rare and only rarely are associated ligamentous injuries. The injury mechanism is commonly considered to be similar with ankle sprains, where excessive varus of the hindfoot leads to avulsion fractures of the lateral process of the talus. However, previous cadaveric studies have suggested that LTPF was more likely to be caused by eversion or external rotation force with dorsiflexion of the ankle. But no clinical evidence has been provided.

**Case presentation:**

Two patients presented to the emergency department with ankle pain after ankle eversion or external rotation. Physical examination revealed tenderness and swelling on both medial and lateral sides of the ankles. Plain radiographs and computed tomography revealed LTPF and medial soft tissue swelling, and magnetic resonance imaging confirmed a discontinuity of the deltoid ligament in Case 1. Surgical exploration revealed rupture of the superficial layer of the deltoid ligaments with intact deep layer in both patients. Treatment included fixation of the lateral process of the talus with headless compression screws and repair of deltoid ligaments. Both patients achieved excellent clinical outcomes 1 year post injury.

**Conclusion:**

There are many possibilities of the injury mechanism of LTPF. These two cases provided clinical evidence that eversion or external rotation force, in addition to inversion, was also an important mechanism of LTPF.

**Supplementary Information:**

The online version contains supplementary material available at 10.1186/s12893-022-01781-y.

## Background

Fractures of the lateral process of the talus (LTPF), also known as “snowboarder’s ankle”, are very rare in the general population [1]. Of all fractures, 0.1–0.85% are talar fractures and only 10.4–20% of these involve the lateral process [2, 3]. Most LTPF are isolated fractures and only rarely are associated ligamentous injuries [4]. The mechanism of this injury is traditionally presumed to be forced dorsiflexion of the foot with associated inversion that lead to avulsion fracture of the lateral process of the talus [5, 6]. In some cases, the calcaneofibular ligaments have also been reported to be ruptured [7]. However, some cadaveric biomechanical studies proposed that LTPFs could also be caused by dorsiflexion of the foot with associated eversion or external rotation [8]. To our knowledge, little clinical evidence has been provided to support this injury mechanism. In this study, we describe 2 cases of LTPF with associated deltoid ligament injury, which provides indirect evidence on the possibility of the eversion/external rotation injury mechanism.

## Case presentation

### Case 1

**Fig. 1:**
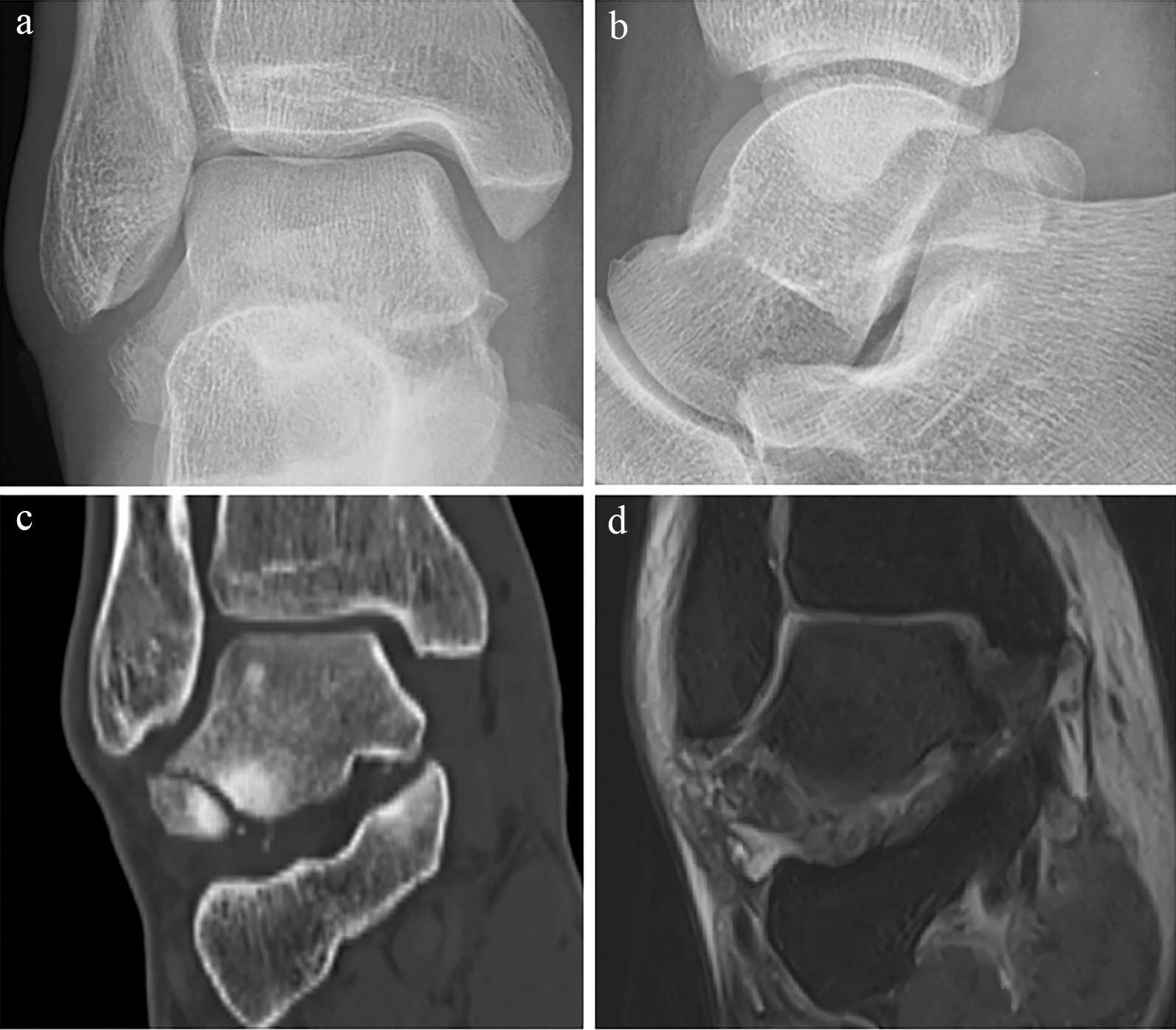
The preoperative imaging data of case 1. **a** Anteroposterior and **b** lateral radiograph of the injured ankle showed obscure fracture lines of the lateral process of the talus. **c** CT scan revealed fracture of the lateral process of the talus as well as soft tissue swelling around the medial malleolus. **d** MRI using fat-suppressed proton-density turbo-spin-echo showed high signal around the superficial deltoid ligament and mixed signal of the deep deltoid ligament

A 29-year-old man who presented to the emergency department after a bicycle accident described an external rotation injury to his right ankle when the bicycle fell on his foot. Physical examination revealed tenderness and swelling on the medial and lateral ankle joints. Plain radiographs and computed tomography (CT) scans were performed, showing a slightly displaced fracture of the lateral talus (Fig. 1a–c). Magnetic resonance imaging (MRI) using fat-suppressed proton density turbo spin echo (FS PD-TSE) showed hyperintensity around the superficial layer of the deltoid ligament, and mixed signal deep in the deltoid ligament (Fig. 1d), indicating a potential rupture of the deltoid ligament (Additional file 1). Four days after injury, the patient underwent surgical fixation of the lateral talus and deltoid ligament exploration.

### Case 2

**Fig. 2:**
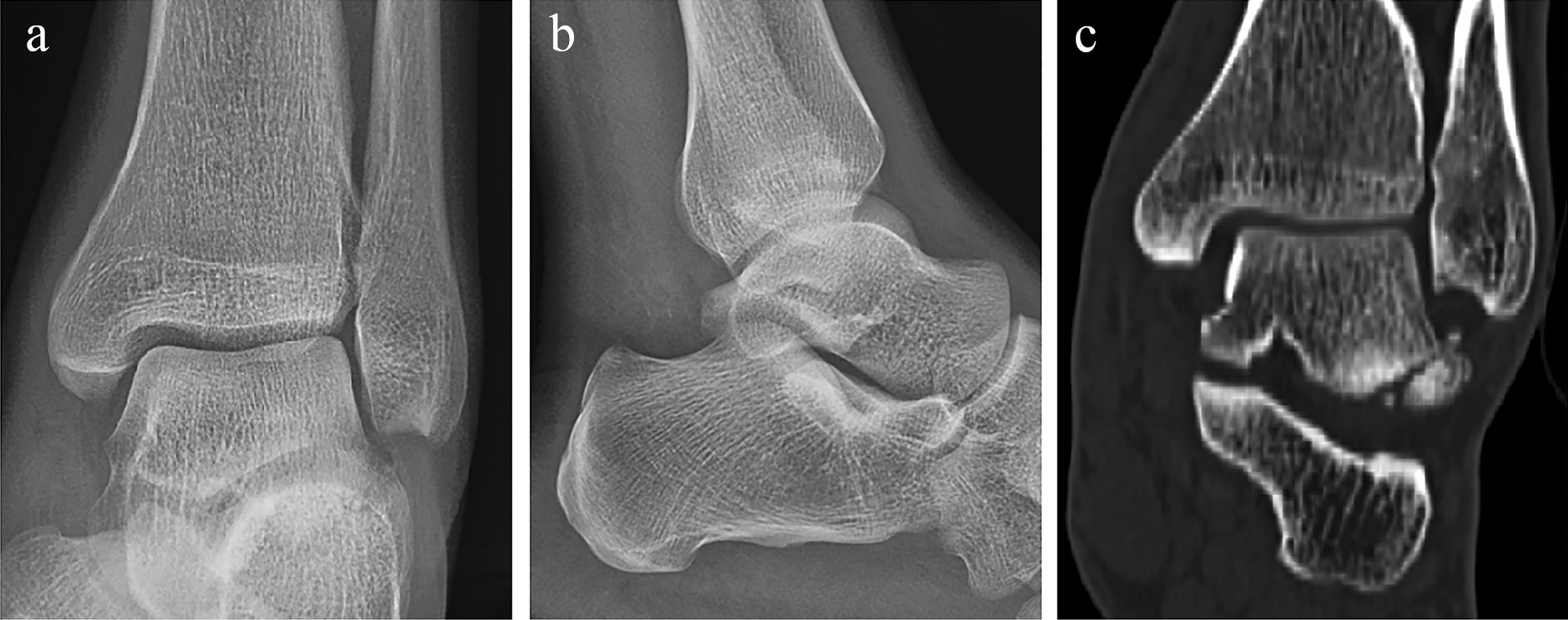
The preoperative imaging data of case 2. **a** Anteroposterior and **b** lateral radiograph of the injured ankle showed no obvious fracture of the ankle. **c** Computed tomography scan revealed fracture of the lateral process of the talus with several chip fragments on both fibular and calcaneal articular surfaces of the talus. On the medial side, soft tissue swelling could be observed around the malleolus medialis

A 36-year-old male elevator mechanic who presented to the emergency room immediately after a labor accident described a left ankle valgus injury. Physical examination revealed severe swelling and ecchymosis on the medial side of the left ankle, as well as distal tenderness of the distal fibula. Plain radiographs and CT were performed immediately, showing LTPF and medial soft tissue thickening of the left ankle (Fig. 2). When soft tissue swelling subsided, surgery was delayed until 8 days after injury.

### Operative technique

**Fig. 3:**
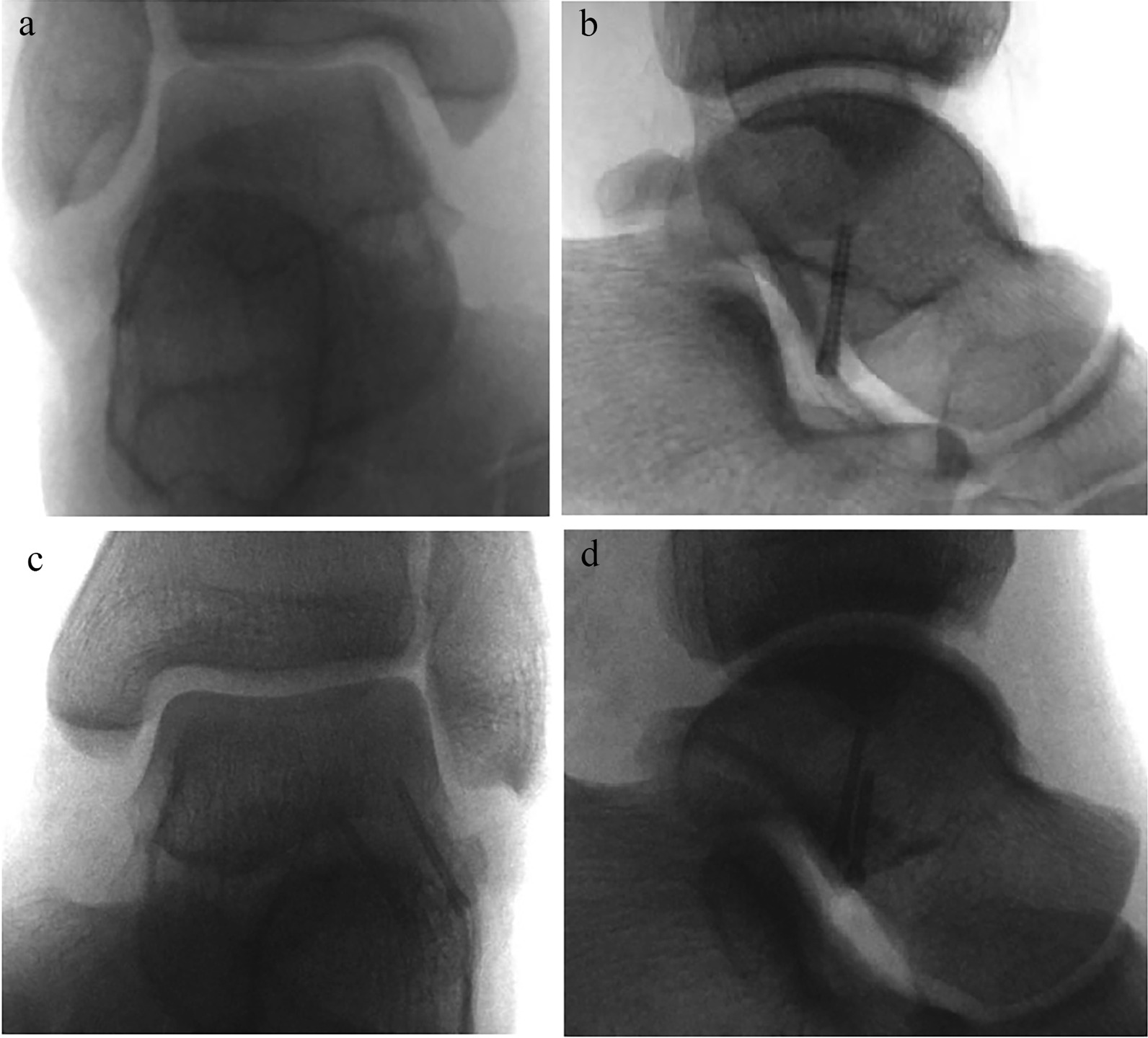
Intraoperative fluoroscopic images of the two cases after screw fixation of the lateral process of talus. Case 1: (**a**) Anteroposterior and (**b**) lateral radiograph showed screw fixation with one headless compression screw. Case 2: (**c**) Anteroposterior and (**d**) lateral radiograph showed screw fixation with two headless compression screws

Both patients underwent surgical fixation of the lateral process of the talus and surgical exploration of the deltoid ligament with similar operative technique. The operation was performed in a supine position under general anesthesia. Prophylactic antibiotics were administered half an hour before the operation. Firstly, a slightly curved incision of 6 cm was made on the lateral side, starting at the base of the fourth metatarsal and ending at the posterior and inferior margin of the fibula. After division of subcutaneous tissue, the inferior extensor retinaculum and the capsule were incised over the peroneal tendon to expose the fracture site. Blood clots and small chip fragments at the fracture site were removed, while the main fragment was retained and temporarily fixed using 1.2-mm Kirschner wires. Under the confirmation of fluoroscopy, one or two 2.0-mm cannulated screws were inserted for definitive fixation (Fig. 3).

**Fig. 4:**
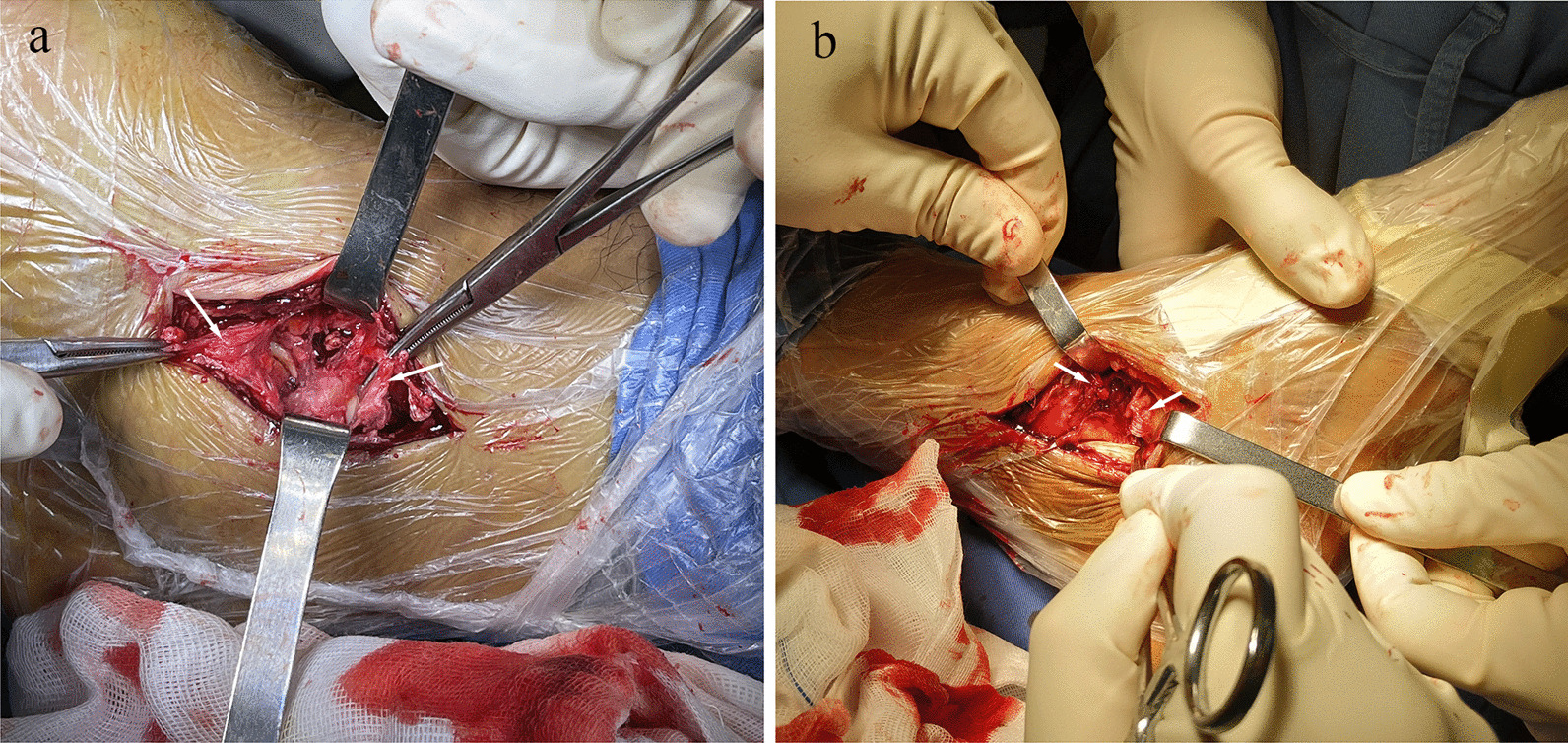
Intraoperative photographs of the deltoid ligament rupture of the two cases. In both (**a**) case 1 and (**b**) case 2, superficial deltoid ligaments were ruptured at the middle portion. The deep layer of the deltoid ligaments was examined with a probe and proved to be intact. White arrow: Torn ends of the deltoid ligaments

On the medial side, a posteromedial incision was made from the tubercle of the tarsal navicular bone to the posterior border of the medial malleolus. After division of the subcutaneous layer, the flexor retinaculum was found to be intact in both cases. Following the longitudinal incision of the flexor retinaculum, the superficial deltoid ligament was exposed and found to be ruptured in the middle portion in both cases (Fig. 4). The integrity of the deep deltoid ligament was examined with a probe and proved to be intact. Then, the superficial layer of the deltoid ligament was repaired, followed by the flexor retinaculum. Finally, the subcutaneous layer and the skin were closed.

### Postoperative rehabilitation

For the first 6 weeks, the ankle was kept in a neutral position with an under-knee cast and partial weight bearing was allowed up to 10 kg. Afterwards, loading was gradually increased so that after 8 weeks full weight bearing was allowed. Meanwhile, the patients were encouraged to perform rehabilitation exercises including ankle plantar flexion, dorsiflexion, inversion, and eversion with a resistance band, aiming to restore range of motion and improve muscular strength.

One year after surgery, both patients restored pain-free walking and returned to pre-injury level of activity. They were satisfied with the results, with the mean American Orthopedic Foot and Ankle Association Score being 92.

## Discussion

Once a rare injury, LTPF has recently been on the rise with the popularity of snowboarding [9]. However, the injury mechanism of LTPF remains unclear. Early reports suggested that LTPF and ankle sprain shared similar symptoms and injury mechanism, namely forced inversion of the foot [1, 5, 6, 10, 11]. The anatomical basis of the inversion mechanism is that the talofibular and talocalcaneal ligaments terminate in the lateral process of the talus. Therefore, an inversion force could cause overstretching of the lateral talocalcaneal ligament, tearing the lateral process from the talus. This theory provided further evidence that that LTPF could occur with concomitant lateral ligaments injury [7].

However, some authors proposed that LTPF could also be a result of an eversion or external rotation force toward the foot. In a previous cadaveric study, Boon et al. [12] failed to create LTPF with a combined force of axial loading, dorsiflexion, and inversion. But with additional external rotation, six out of eight specimens sustained LTPF. A similar cadaveric study by Funk et al. [13] demonstrated that all the specimens subjected to eversion force sustained LTPF. However, little clinical evidence has yet been provided. The two cases in this study were the first to describe LTPF with associated deltoid ligament injury. In these two cases, the superficial deltoid ligaments were ruptured, implying hindfoot eversion or external rotation might be the primary injury mechanism in this combined injury [14].

Due to the low incidence, there has been no clear surgical indication of LTPF. McCrory and Bladin [1] classified LTPF into three types: type I is a nonarticular chip fracture, type II is a single large fragment involving both talofibular and subtalar joint, and type III is a comminuted fracture. In general, conservative treatment is advised for type I fractures and surgical treatment is usually addressed for type II and type III fractures. Perera et al. [15] reviewed previous studies and discovered that patients with McCrory-Bladin type II LTPF treated operatively did much better than those treated conservatively. Those who were treated conservatively had a 38% chance of moderate or severe symptoms and 47% required later surgery [15]. In another study, Ross et al. [16] reported a failure rate of 67% after conservative treatment and concluded that patients treated with early surgery have significantly fewer subtalar fusions as compared to patients with nonoperative treatment. Only nondisplaced, small-fragment, and extra-articular fractures could be treated conservatively [17].

As for the treatment of deltoid ligament, no clear indications for operative repair have been well established in the current literature [18, 19]. In this study, surgical exploration of the deltoid ligaments was applied to reveal the pathologic changes in this combined injury and ligament repair was done afterwards. However, we do not encourage deltoid ligament repair as a routine procedure in future studies since the deep layer is most likely to be intact, and recent studies show that deltoid ligament repair does not significantly improve function in ankle fractures [19].

## Conclusion

There are many possibilities of the injury mechanism of LTPF. These two cases provided clinical evidence that eversion or external rotation force, in addition to inversion, was also an important mechanism of LTPF.

## Supplementary Information

**Additional file 1. **Multiplanar reconstruction of MRI using FS PD-TSE sequence of Case 1.

## Data Availability

The datasets used and/or analysed during the current study are available from the corresponding author on reasonable request.

## Abbreviations

LTPF: Fractures of the lateral process of the talus
CT: Computed tomography
MRI: Magnetic resonance imaging
FS PD-TSE: Fat-suppressed proton-density turbo-spin-echo
