# Supplementary figures and images for: Fracture of the lateral process of the talus with associated deltoid ligament injury: a report of 2 cases
Source: BMC Surg. 2022 Oct 4;22:356. doi: 10.1186/s12893-022-01781-y (PMC9533565; doi:10.1186/s12893-022-01781-y)

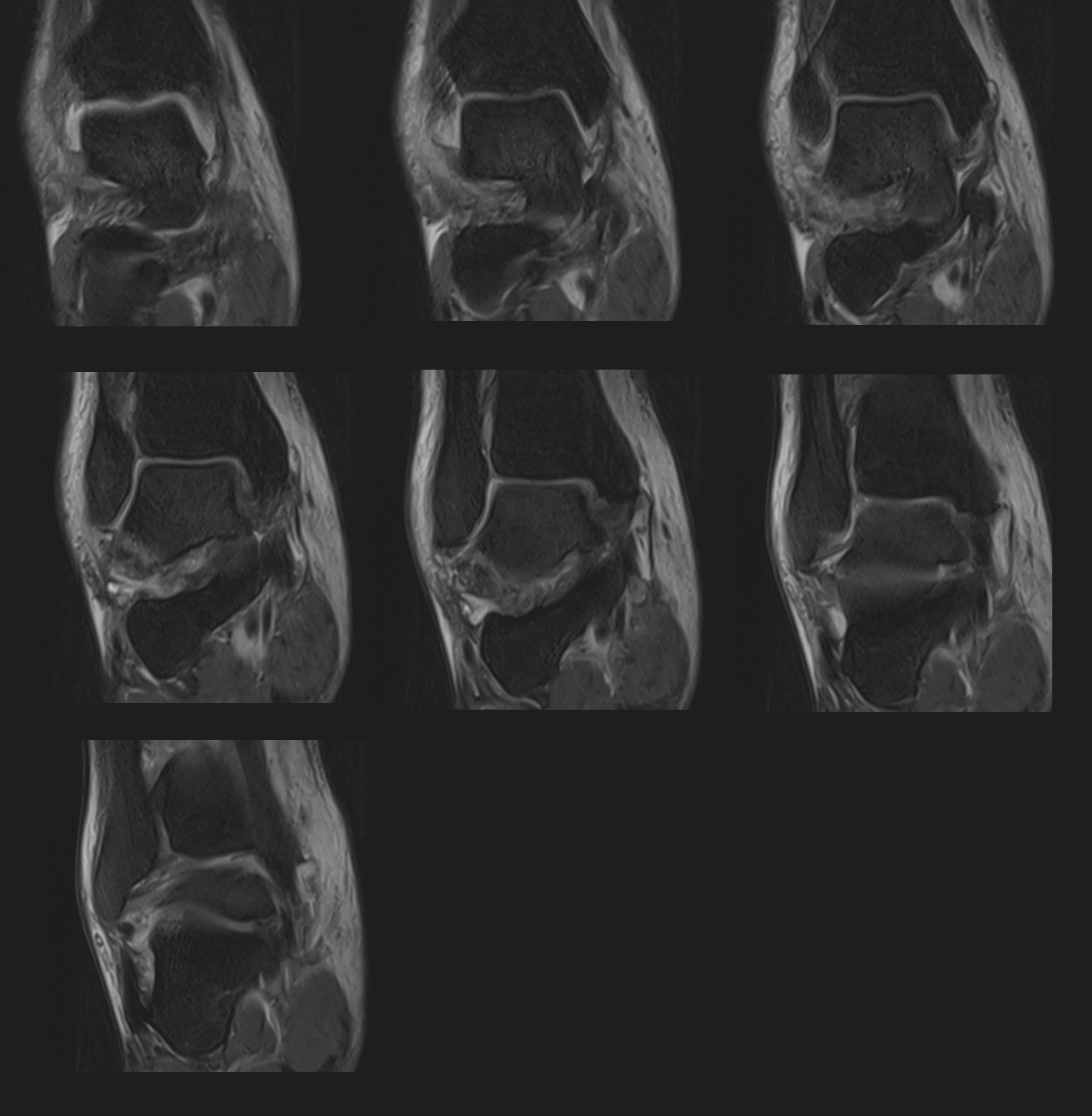

Supplement: Supplementary file 1 — Additional file 1. Multiplanar reconstruction of MRI using FS PD-TSE sequence of Case 1. [file 12893_2022_1781_MOESM1_ESM.tif]
